# Terrestrial nematodes from the Maritime Antarctic

**DOI:** 10.3897/BDJ.11.e102057

**Published:** 2023-09-14

**Authors:** Milka Elshishka, Aleksandar Mladenov, Stela Lazarova, Vlada Peneva

**Affiliations:** 1 Institute of Biodiversity and Ecosystem Research, Bulgarian Academy of Sciences, 2 Gagarin Street, 1113, Sofia, Bulgaria Institute of Biodiversity and Ecosystem Research, Bulgarian Academy of Sciences, 2 Gagarin Street, 1113 Sofia Bulgaria

**Keywords:** endemics, distribution, DNA sequences, species

## Abstract

**Background:**

Soil nematodes are one of the most important terrestrial faunal groups in Antarctica, as they are a major component of soil micro-food webs. Despite their crucial role in soil processes, knowledge of their species diversity and distribution is still incomplete. Taxonomic studies of Antarctic nematodes are fragmented, which prevents assessment of the degree of endemicity and distribution of the species, as well as other aspects of biogeography.

**New information:**

The present study is focused on the nematode fauna of one of the three Antarctic sub-regions, the Maritime Antarctic and summarises all findings published up to April 2023. A species list that includes 44 species, belonging to 21 genera, 16 families and eight orders is provided. A review of the literature on terrestrial nematodes inhabiting the Maritime Antarctic showed that the sites are unevenly studied. Three islands (Signy, King George and Livingston Islands) revealed highest species richness, probably due to the highest rates of research effort. Most species and four genera (*Antarctenchus*, *Pararhyssocolpus*, *Amblydorylaimus* and *Enchodeloides*) are endemic, proving that nematode fauna of the Maritime Antarctic is autochthonous and unique. Several groups of islands/sites have been revealed, based on their nematode fauna. The study showed that species with a limited distribution prevailed, while only two species (*Plectusantarcticus* and *Coomansusgerlachei*) have been found in more than 50% of the sites. Based on the literature data, details on species localities, microhabitat distribution, plant associations and availability of DNA sequences are provided.

## Introduction

Soil nematodes are one of the most important groups of the terrestrial fauna in Antarctica (Maslen and Convey 2006) as they are abundant, taxonomically and functionally diverse and occupy a central position in the soil micro-food webs (Adams et al. 2014) and may have an impact on nutrient cycling and carbon dioxide emission, when soils thaw for a longer period of the year under climate change (van den Hoogen et al. 2019). In the challenging environmental conditions of the Antarctic, their distribution is limited to ice-free areas, where they have evolved throughout millions of years of climatic fluctuations in refugia (Ebach et al. 2008, Convey et al. 2020, Stevens and Mackintosh 2023). The glaciations, long-term isolation, harsh climate and the patchy distribution of ice-free areas (present today where at least partially ice-free throughout repeated glacial maxima (Newman et al. 2009)) are the main factors affecting the Antarctic nematode fauna origin/genesis (i.e. the formation of fauna under the influence of multiple factors - historical, geographic and ecological) (Andrássy 1998, Convey and Peck 2019). In order to survive in the extreme environments, nematodes have developed exceptional cryptobiotic adaptations to manage freezing and desiccation stress (e.g. Pickup (1988), Wharton (1995), Treonis and Wall (2005), Kagoshima et al. (2019)).

Knowledge of the impact of climate change on nematode communities from extreme habitats and how they respond to these changes is insufficient (Freckman and Virginia 1997, Nielsen et al. 2011a). One of the main problems in predicting the effects of climate change in Antarctica is the limited knowledge on the diversity of terrestrial fauna, especially nematodes and the lack of comprehensive long-term studies (Gantait 2014). Data on species distribution and biogeography are not enough and the taxonomic information still remains confused or scarce (Andrássy 1998, Maslen and Convey 2006, Adams et al. 2014, Kagoshima et al. 2019). Nematodes possess high indicator potential for assessing various environmental changes in the soil environment because they are abundant, ubiquitous, utilise diverse trophic and live strategies and, thus, occupy key positions in soil micro-food webs (Neher 2001, Ferris et al. 2001, Neher 2010, Chauvin et al. 2020, Taylor et al. 2020, Ara Khanum et al. 2022, Du Preez et al. 2022). This highlights the need for research on the fauna of nematodes and their communities in extreme environments in view of the already occurring global change.

Antarctica represents three distinct climatic regions: the Sub-Antarctic, Maritime and Continental Antarctic (Holdgate 1977), with the Sub-Antarctic being the most favourable (mean air temperatures of most islands are low, but positive during the whole year), with the Continental Antarctic having the harshest conditions (the average monthly temperatures remain below freezing) (Convey 2017). The Continental Antarctic covers the territories of the continent, the Balleny Islands and the eastern side of the Antarctic Peninsula (Convey 2017). The Sub-Antarctic is the boundary zone that lies north of 56°S (Chown and Brooks 2019). The flora and fauna in this region are rather typical of temperate latitudes. In this paper, we focused on terrestrial nematodes from the Maritime Antarctic. This is a region with a strong influence of the Southern Ocean; it includes the western coast of the Antarctic Peninsula to ca. 72°S, the South Shetland, South Orkney and South Sandwich Islands and the isolated Bouvetøya and Peter I Øya (Convey 2006, Convey 2017). The Maritime Antarctic is characterised by more favourable conditions compared with the Continental Antarctic: mean air temperatures are positive for 1–4 months of the year (Convey 2017), the vegetation is predominantly cryptogamic (algae, mosses, liverworts, lichens); higher plants are represented by two species, *Deschampsiaantarctica* Desv. (Poaceae) and *Colobanthusquitensis* Bartl. (Caryophyllaceae) (Greene 1970, Longton 1979, Smith 1984).

Studies on nematodes in the Maritime Antarctic started at the beginning of the 20^th^ century, with the contribution of the Romanian biologist Emil Racoviţă during the first scientific Antarctic expedition in winter (Andrássy 1998). This resulted in a description of the first Antarctic nematode species, *Mononchusgerlachei* (= *Coomansusgerlachei* (de Man 1904) Jairajpuri and Khan 1977), followed by three other species, *Plectusantarcticus de Man 1904*, *P.belgicae* de Man 1904 and *Dorylaimus* sp. Following these first data, targeted investigations on the terrestrial nematode fauna from this part of Antarctica started from the early 70s of the 20^th^ century (Spaull 1972, Spaull 1973a, Spaull 1973b, Spaull 1973c, Loof 1975, Maslen 1979a, Maslen 1979b, Caldwell 1981, Maslen 1981, Spaull 1981, Pickup 1988, Shishida and Ohyama 1989, Tsalolikhin 1989, Pickup 1990, Janiec 1996, Peneva et al. 1996, Andrássy 1998, Peneva and Chipev 1999, Convey et al. 2000, Nedelchev and Peneva 2000, Peneva et al. 2002, Convey and Wynn-Williams 2002, Holovachov and Bostrom 2006, Maslen and Convey 2006, Nedelchev and Peneva 2007, Kito 2009, Peneva et al. 2009, Nielsen et al. 2011b, Velasco-Castrillón and Stevens 2014, Velasco-Castrillón et al. 2014a, Russell et al. 2014, Elshishka et al. 2015a, Elshishka et al. 2015b, Elshishka et al. 2017, Kagoshima et al. 2019, Newsham et al. 2020). Antarctic nematodes have been studied mainly in easy to access areas near to the research bases/stations; therefore, there are still many remote locations never sampled for nematodes which raise questions on how widespread the species are (Adams et al. 2014, Convey et al. 2020).

According to Andrássy (1998), numerous studies have reported species as new records with no morphological description making it impossible to confirm identifications, especially when the collected material is no longer available for subsequent examination. Further, this has an impact on the potential to assess fauna endemicity, which is critical for examining Antarctic biogeography within a global context (Andrássy 1998). There are numerous cases of misclassification and underestimation of the diversity for most microfaunal groups in Antarctica, likely due to poor taxonomic resolution caused by insufficient sampling and their difficult identification (Adams et al. 2006, Iakovenko et al. 2015, Carapelli et al. 2017, Short et al. 2022, Collins et al. 2023), as well as the low degree of the development and application of molecular taxonomy.

In recent years, molecular studies have become more important in these marginal habitats, as a powerful toolkit to complement the traditional taxonomy, species identification and descriptions and to assess biodiversity and biogeography (Courtright et al. 2000, Velasco-Castrillón et al. 2014b, Elshishka et al. 2015b, Elshishka et al. 2017, Czechowski et al. 2017, Velasco-Castrillón et al. 2018, Kagoshima et al. 2019).

The integrative approach (combining morphological and molecular data) is an effective way to understand the scale of endemism, evolution and distribution of the Antarctic nematode fauna. However, the main problem of not linking molecular data with morphology still remains for the vast majority of Antarctic nematode species.

The present paper aims to summarise all records of nematode species occurrence in the Maritime Antarctic between the years of 1904 and April 2023 as a basis for further studies and to present a snapshot of nematode species diversity in this part of the Antarctic.

## Materials and methods

The nematode species list has been composed, based on literature data and refers to the Maritime Antarctic. This list includes all species recovered in the Maritime Antarctic, as well as the islands and sites from where each species was reported, along with data on microhabitats and plant associations, accession numbers of published sequences in GenBank also included, if available. The type of microhabitat is reported as in the original paper, the scientific names of the plants being adapted according to the current systematics (Ochyra 1998). Geographical coordinates are presented additionally for each site if missing in the original paper. For the literature search, online bibliography search engine Google Scholar and the academic databases Scopus, Web of Science and CABI were used with search keywords “terrestrial nematode species*” and “Maritime Antarctic*”. We focused on studies reporting nematode species (see Holovachov (2014a)) from the Maritime Antarctic and omitted those that provide data only at the generic or family level.

Several papers recording multiple unidentified taxa at generic level (Maslen and Convey 2006, Nielsen et al. 2011b) probably contain many undescribed nematode species from those regions suggesting that the nematode diversity there might be underestimated to a great extent. Overall, nematodes from 37 sites (34 islands and three localities on the Antarctic Peninsula) are included in the review. The taxonomic position of the Antarctic species was presented according to the current nematode nomenclature. Classification follows Andrássy (2005), Andrássy (2007) and Andrássy (2009); only for order Plectida classification follows Holovachov (2014b). The analyses are based on species presence/absence data and Wizard > Matrix display function in PRIMER v.7.0 software (Clarke and Gorley 2015). The Matrix display wizard performs a sequence of sample and species resemblance calculations and clustering and seriation steps resulting in a shade plot which visualises the species presence/absence data and sites similarity.

## Checklists

### Checklist of terrestrial nematodes from the Maritime Antarctic

#### Dorylaimida

DB2E6DDD-5A83-5FAD-AA05-491530706589

#### Nordiidae

02036AD4-B115-5ACB-9D3A-C365D4FFFBE3

#### Enchodeloides signyensis

(Loof, 1975) Elshishka, Lazarova, Radoslavov, Hristov, Peneva, 2017

F37166B3-37C5-55AA-A12F-64A130AA896F

Enchodelus signyensis Loof, 1975

#### Qudsianematidae

15105F37-9676-56CF-887B-328D15B98C4B

#### Eudorylaimus coniceps

Loof, 1975

181A5CE6-3861-500E-A087-F0FF38D50423

#### Eudorylaimus pseudocarteri

Loof, 1975

47CAF4A1-E28A-5E59-ACEB-31BCAA37A957

#### Eudorylaimus spaulli

Loof, 1975

5F4D2E55-53D8-538D-B503-A78CEEA105CE

#### Eudorylaimus verrucosus

Loof, 1975

3FB9DFB0-EEBC-545E-9436-944D9DBBEED3

#### Eudorylaimus cf. carteri

Andrassy, 1959 (Bastian, 1865)

13B2261E-A584-5999-BED0-190056CB4892

#### Pararhyssocolpidae

6C015E2C-BEF0-56CA-AF5D-8AE0D3404203

#### Pararhyssocolpus paradoxus

(Loof, 1975) Elshishka, Lazarova, Radoslavov, Hristov, Peneva, 2015

D9585A55-9B26-58B4-9516-DDC1BC9EB469

Eudorylaimus paradoxus Loof, 1975|Rhyssocolpus paradoxus (Loof, 1975) Andrássy, 1986

#### Dorylaimidae

671787FE-3946-5420-9619-FACA9D2FA213

#### Calcaridorylaimus signatus

(Loof, 1975) Andrássy, 1981

F5A9037F-584C-56F6-A44A-63714ACBC6D2

Mesodorylaimus signatus Loof, 1975

#### Mesodorylaimus antarcticus

Nedelchev and Peneva, 2000

EE444A29-4179-5887-B2A1-F41ADCCD81D6

#### Mesodorylaimus chipevi

Nedelchev and Peneva, 2000

9CD6CDD3-2EF8-5BAE-9D2A-2BCBC01ADE36

#### Mesodorylaimus imperator

Loof, 1975

9515D5C8-859D-5A66-A87E-9B42936469F3

#### Mesodorylaimus masleni

Nedelchev and Peneva, 2000

2A2130FE-3C7C-5B1D-9ADE-2B200FCE8D50

#### Aporcelaimidae

75CC4343-89B8-5900-94FC-936112411653

#### Amblydorylaimus isokaryon

(Loof, 1975) Andrássy, 1998

B044B3FC-B769-5B49-BA3E-B9BC91BE6581

Eudorylaimus isokaryon Loof, 1975

#### Aphelenchida

28744625-5C08-5291-98C0-3794406ED313

#### Aphelenchoididae

01714A28-F5E0-5D6B-B774-10899E47322D

#### Aphelenchoides haguei

Maslen, 1979

D4AC79D2-B450-5D13-9DCB-9E7FA2223288

#### Aphelenchoides vaughani

Maslen, 1979

A1301FE0-4F0B-5836-89C5-9246F2546124

#### Laimaphelenchus helicosoma

(Maslen, 1979) Peneva and Chipev, 1999

EFACFF62-976B-52D8-9526-5BA5A58C10E2

Aphelenchoides helicosoma Maslen, 1979

#### Alaimida

ADEC0201-921A-524A-B652-7B828938C15C

#### Amphidelidae

CBF633E8-7E83-5A54-A021-9C64861B55ED

#### Paramphidelus antarcticus

Tsalolikhin, 1989

D52EDC6A-5219-5C6E-A1F5-5F16A09027F6

#### Monhysterida

6748B1A9-7801-5159-8BB2-F159B2314D3E

#### Monhysteridae

7F94800F-CCCC-5B15-A41F-FBDDAE8C6CF3

#### Eumonhystera filiformis

(Bastian, 1865) Andrássy, 1981

87224B84-E25A-58B9-8B24-D9603402ED4E

#### Eumonhystera vulgaris

(de Man, 1880) Andrássy, 1981

33E17069-31EC-5865-A580-6DC42A82B918

#### Geomonhystera villosa

(Bütschli, 1873) Andrássy, 1981

6766505C-8E58-5AA4-BA98-A18390B2F1C0

#### Plectida

195B637E-A81D-5EC0-9837-4C8317666230

#### Plectidae

02148983-E217-5C83-8D3D-B5BA9EBF4DCD

#### Plectus antarcticus

de Man, 1904

E558E15A-D114-5D9C-BA37-37BC230AD109

#### Plectus cf. antarcticus

de Man, 1904

DF1FF3FE-15D0-5BDB-AFEC-B84A616E61AB

#### Plectus belgicae

de Man, 1904

49B0CF61-2DA8-5A0A-976F-96025CF7414C

#### Plectus cf. belgicae

de Man, 1904

A7BC5E1E-478B-5A29-8CE2-A308A5ABA7F0

#### Plectus insolens

Andrássy, 1998

ECAB5F55-AB36-5933-9BB3-4876420207EA

#### Plectus tolerans

Andrássy, 1998

06295DAB-BBB3-5019-A2F7-0C9B80497144

#### Plectus cf. tolerans

Andrássy, 1998

A9D39BED-6A38-5246-BBC1-7E7217EAE2F3

#### Plectus meridianus

Andrássy, 1998

BC659854-2A25-5484-9336-20F9490DBAC9

#### Plectus cf. meridianus

Andrássy, 1998

976487C9-C3EB-5CF8-B924-F0D5E86B7FB4

#### Plectus armatus

Bütschli, 1873

E97AA054-492C-5A21-B20B-198B5E58EB7B

Ceratoplectus armatus (Bütschli, 1873) Andrássy, 1984

#### Tylenchida

266FDC27-9624-596A-AD7A-24842C567356

#### Psilenchidae

A315F1BF-3304-582D-BB2C-CC87EF8EFC52

#### Antarctenchus hooperi

Spaull, 1972

8412463F-2F6C-5DB8-AF9A-7E4457E73FC2

#### Anguinidae

8DA64EBF-3D67-5C57-A8D9-9D104D5B8277

#### Ditylenchus parcevivens

Andrássy, 1998

E2215FCD-DAF8-5AB4-819E-30C2BA885C08

#### Rhabditida

51F7A518-984C-5256-93F6-F883548EFB18

#### Teratocephalidae

CDEA95B0-7F13-5883-9856-2C609C4E06F1

#### Teratocephalus tilbrooki

Maslen, 1979

3B584363-B128-5856-A2DB-DC6BDB952CA7

#### Teratocephalus pseudolirellus

Maslen, 1979

0BEC9F2C-E0DD-54EF-A34F-27716F289B59

#### Teratocephalus rugosus

Maslen, 1979

AD7D6030-A3BD-5870-A253-9F5E9C120EA1

#### Cephalobidae

82D975B4-05C7-5984-AD81-AAFC584A518B

#### Acrobeloides arctowskii

Holovachov and Boström, 2006

7C694E1F-086F-590F-9BBB-EF082420FBE7

#### Cervidellus cf. vexilliger

(de Man, 1880) Thorne, 1937

DE9D79DC-B582-5B55-B83A-1485B5BF627C

#### Rhabditidae

58F28B03-4A75-5365-B10C-05773A48BEB6

#### Cuticularia firmata

Andrássy, 1998

107613C0-FDBC-560E-9D60-60066C61F32B

#### Rhabditis krylovi

Tsalolikhin, 1989

D26C2B68-3D22-5133-A37C-DB9F260A2761

#### Rhabditis marina -group

410838AE-0F98-5B3C-AE3E-0CCB5F4D407A

#### Peloderidae

8C42EF21-19F7-5F43-A2E3-1A7D323A102B

#### Pelodera teres -group

F43B566C-DE04-54B9-881E-D65B10FA9A59

#### Pelodera strongyloides -group

C5A2BEFB-7A41-50C1-9050-C7E4AC3EA8F9

#### Pelodera parateres -group

D1D7791E-0B8D-5994-A75E-6FF0B8A08B8F

#### Mononchida

417FEFF6-89BD-5372-82DA-295F4AF64FC8

#### Mononchidae

CCA60FEF-1D5F-5DB5-A53E-25427B4590F4

#### Coomansus gerlachei

(de Man, 1904) Jairajpuri and Khan, 1977

4DDB6E9C-AEB1-54D8-95B5-28D16F93D36D

Mononchus gerlachei de Man, 1904|*Clarkusgerlachei* (de Man, 1904) Jairajpuri, 1970

## Analysis

### Results

**Figure 1.**
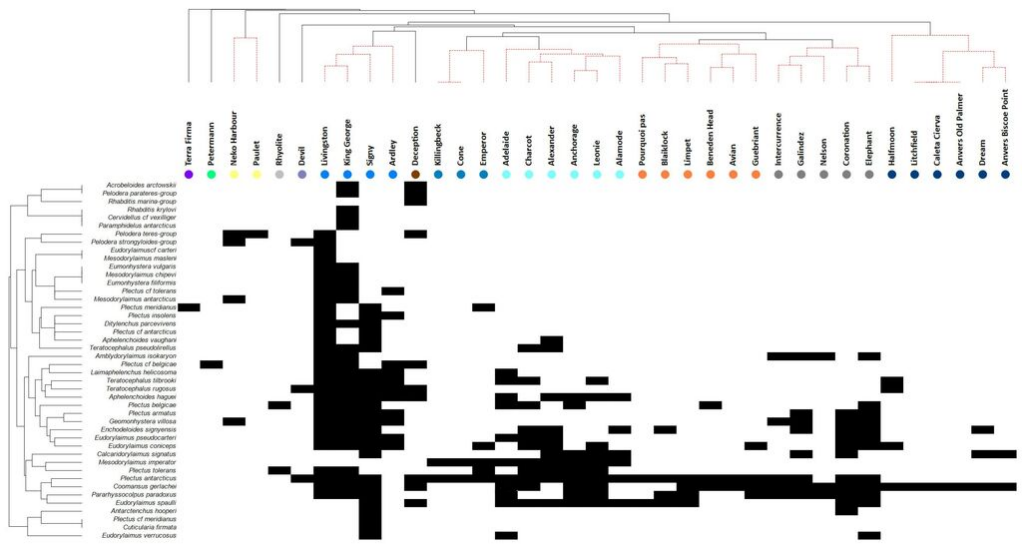
Terrestrial nematodes from the Maritime Antarctic - visual representation of the data matrix (shade plot): in the columns are the 37 sites and in the rows – 44 species. White and black spaces denote absence or presence of a particular species at a given site; sites and species are arranged according to the groups derived by the clustering analyses. Significant clusters were identified with SIMPROF test and visualised in red dashed lines and a range of coloured dots. Each colour represents a group of sites/islands with similar nematode fauna.

**Figure 2.**
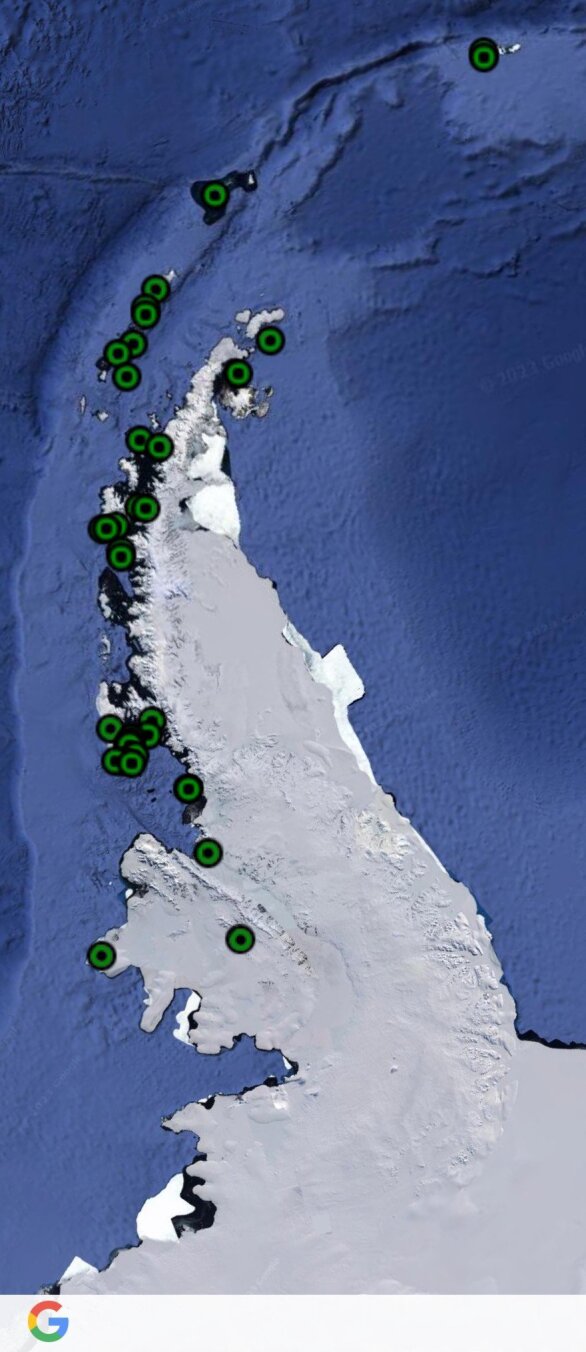
Distribution of terrestrial nematodes in the Maritime Antarctic. In green are presented the sites with records of terrestrial nematodes.

To date, 44 species of terrestrial nematodes, belonging to 21 genera, 16 families and eight orders have been recorded in the Maritime Antarctic (Table 1, Fig. 1). Nematodes have been reported from 34 islands and three sites on the Antarctic Peninsula (Fig. 2). Several groups of islands/sites have been revealed, based on their nematode fauna. Those groups form a gradient from north (the group of Livingston, King George and Signy Islands) to south (the group of Adelaide, Charcot, Alexander, Leonie and Alamode Islands).

The order Dorylaimida is the best represented order in this Antarctic Region with five families, six genera and 13 species. The order Mononchida is represented by only one family (one genus and species).

The families Aphelenchoididae, Cephalobidae, Monhysteridae, Plectidae, Qudsianematidae, Peloderidae and Rhabditidae have a cosmopolitan distribution and, in the Maritime Antarctic, they are represented by one to two genera and two to ten species. The family Plectidae is the most diverse (10 species). Seven families (Amphidelidae, Anguinidae, Aporcelaimidae, Mononchidae, Nordiidae, Pararhyssocolpidae and Psilenchidae) are represented by only one species each.

Almost all species and four genera (*Antarctenchus*, *Pararhyssocolpus*, *Amblydorylaimus* and *Enchodeloides*) are endemic. Four species generally known as cosmopolitan are reported in some ecological studies in the Maritime Antarctic: *Eumonhysteravulgaris* (de Man 1880) Andrássy (1981), *E.filiformis* (Bastian 1865) Andrássy (1981), *Geomonhysteravillosa* (Bütschli 1873) Andrássy (1981) and *Plectusarmatus Bütschli 1873*. Of these, a description and illustrations were provided only for *E.vulgaris (Tsalolikhin 1989*).

**Figure 3.**
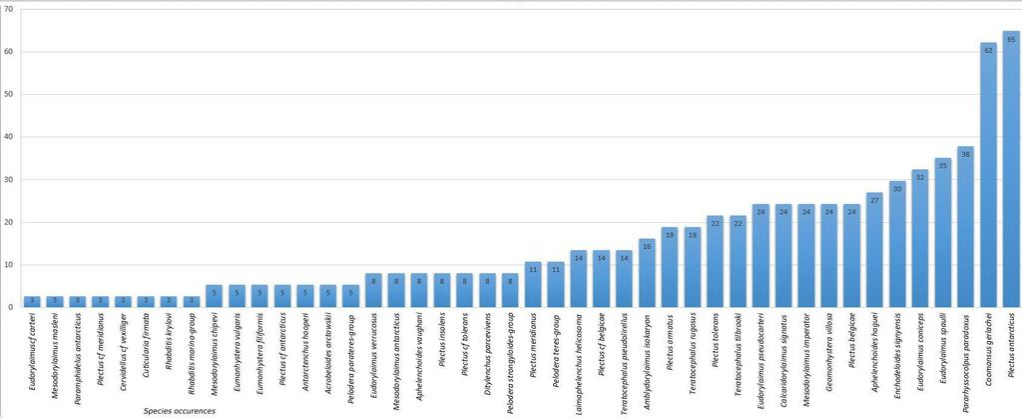
Described species and their occurrences presented as percentages.

**Figure 4.**
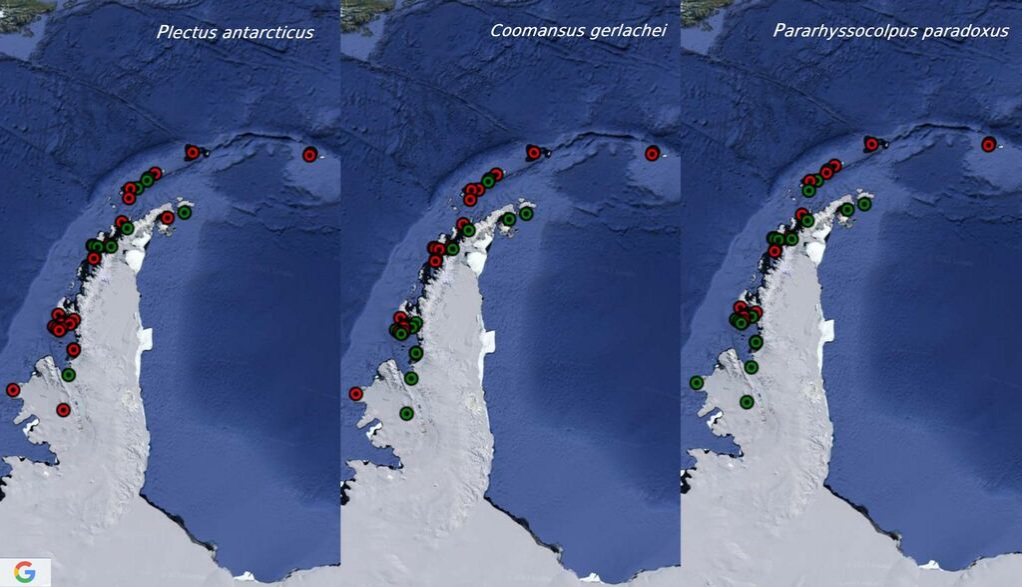
Distribution of *P.antarcticus*, *C.gerlachei* and *Pararhyssocolpusparadoxus* in the Maritime Antarctic. In red are presented the sites with records of these species, in green are presented the sites with records of the other Antarctic terrestrial nematodes.

**Figure 5.**
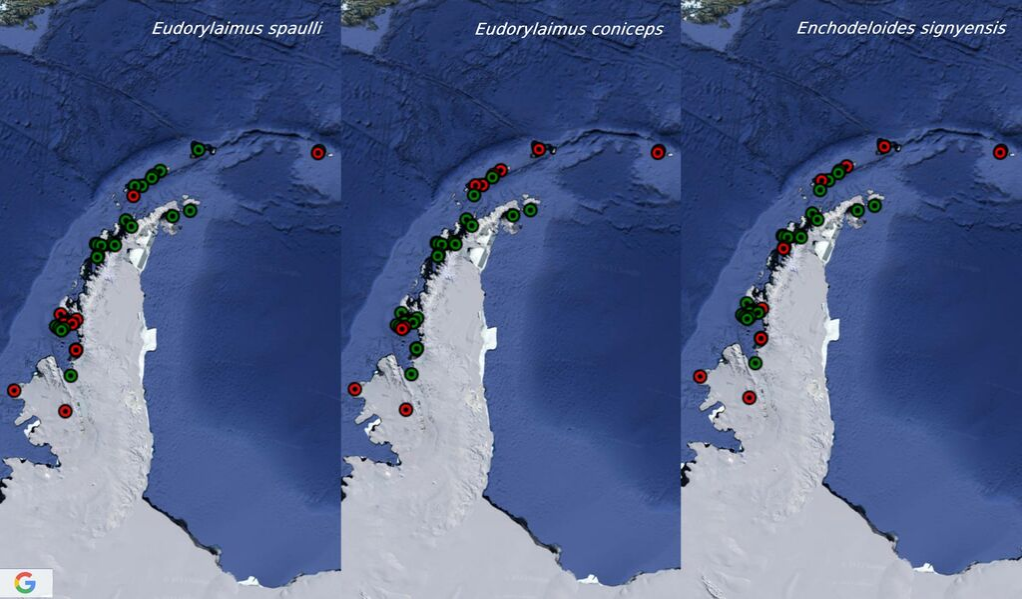
Distribution of *Eudorylaimusspaulli*, *E.coniceps* and *Enchodeloidessignyensis* in the Maritime Antarctic. In red are presented the sites with records of these species, in green are presented the sites with records of the other Antarctic terrestrial nematodes.

Most species (27) have limited distribution registered in up to five islands of the Maritime Antarctic. *Cuticulariafirmata Andrássy 1998*, Cervidelluscf.vexilliger, *Rhabditiskrylovi Tsalolikhin 1989*, a species of the *Rhabditismarina*-group, *Mesodorylaimusmasleni* Nedelchev and Peneva 2000, Eudorylaimuscf.carteri and Plectuscf.meridianus are recorded from one island only. Six species occurred in more than 30% of the sites (*C.gerlachei*, *P.antarcticus*, *Pararhyssocolpusparadoxus* (Loof 1975), *Eudorylaimusspaulli Loof 1975*, *E.coniceps* Loof 1975, *Enchodeloidessignyensis* (Loof 1975)) with *C.gerlachei* and *P.antarcticus* being the most widespread (reported from more than half of the sites) (Figs 3, 4, 5). There are no particular trends in the distribution of most common species (occurring in more than 22% of the sites, 1/4 of the species) related to longitude or latitude, only *P.paradoxus* and *Mesodorylaimusimperator* Loof 1975 have not been reported from the most southern sites, whereas *G.villosa* – from the most northern localities.

**Figure 6.**
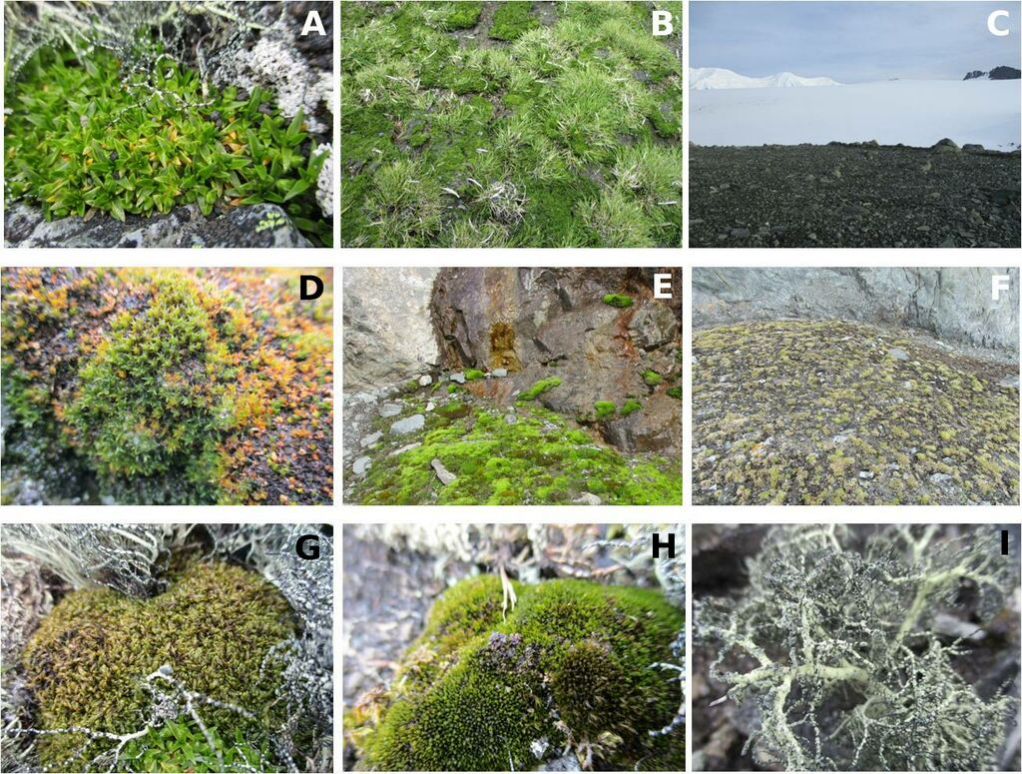
Some microhabitats in the Maritime Antarctic. **A** *Colobanthusquitensis* **B** *Deschampsiaantarctica* **C** Bare soil **D** *Syntrichia* sp. **E** *Bryum* sp. **F** *D.antarctica*, *C.quitensis*, mosses **G** *Sanionia* sp. **H** *Polytrichum* sp. **J** *Usnea* sp. Photographs by M. Elshishka (Livingston Island).

In most of the literature sources, there are data on the microhabitats in which nematode species occurred. The nematodes have been recorded from various microhabitats: bare soil, microbial mats, moss, lichens and algae and soil around the two species of higher plants occurring in the Maritime Antarctic (Fig. 6).

DNA data have been generated for 11 species, but sequences for only three of them (*Amblydorylaimusisokaryon* (Loof 1975), *P.paradoxus* and *E.signyensis*) are supported by full morphological descriptions as per the modern taxonomic standards (Elshishka et al. 2015b, Elshishka et al. 2017, Kagoshima et al. 2019).

**Figure 7.**
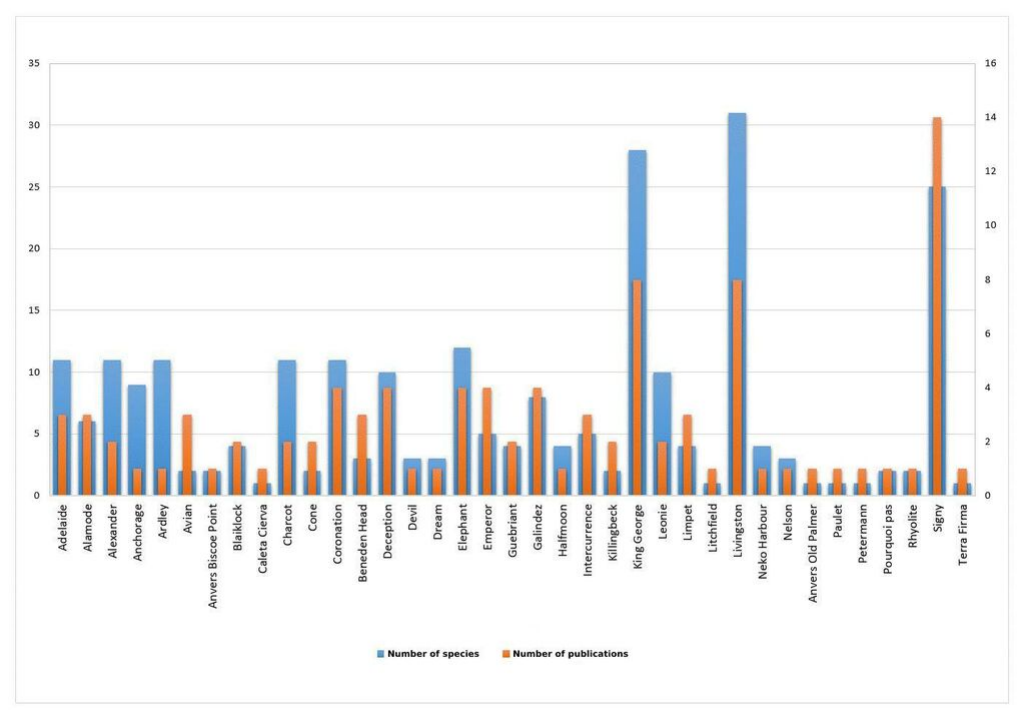
Bar chart visualising the described species (left axis) and literature sources (right axis) per each island/site.

The review of the literature related to terrestrial nematodes from the Maritime Antarctic showed that the different parts are unevenly studied and three islands, Livingston (31 species), King George (28 species) and Signy (25 species) exhibited the richest nematode fauna (Fig. 7). Signy Island is the best studied Antarctic island with 12 new species described. This is due to the intensive studies on the nematode fauna in the 1970s and 1980s undertaken by the British Antarctic Survey (*Spaull 1973a, Spaull 1973b, Spaull 1973c, Loof 1975, Maslen 1979a, Maslen 1979b, Caldwell 1981, Maslen 1981, Pickup 1988, Pickup 1990* etc.).

## Discussion

Our knowledge of the nematode species diversity in the Maritime Antarctic is still insufficient and fragmented. The different study efforts at the various sites do not allow gaining a clear picture of trends in the diversity and distribution of nematode species in the target Antarctic Region. Yet, the analysis provided on the basis of species presence/absence data revealed several groups of sites with similar nematode fauna forming a latitudinal gradient (Fig. 1). The high level of endemism at both the species and genus level is a characteristic feature of the nematode fauna of the region as was mentioned above. This high degree of endemism can be explained by the long-term isolation and the harsh conditions of the region (Convey et al. 2008, Nielsen et al. 2011a). It has been suggested that the Antarctic terrestrial fauna might have survived glaciation in ice-free areas and some species might be remnants of the fauna of the Gondwana super-continent (Andrássy 1998, Maslen and Convey 2006, Chown and Convey 2016, Convey et al. 2020).

The physical isolation and harsh environment of Antarctic terrestrial ecosystems is the major reason for the difficult colonisation by non-native biota (Convey and Peck 2019). In recent decades, human visits and activities in the Antarctic have provided ways (e.g. cargo, vehicles, scientific equipment, fresh food, clothing, people) to overcome these barriers (Lee and Chown 2009, Hughes et al. 2010, Chwedorzewska et al. 2013, Adams et al. 2014). So far, the probability that introduced invertebrates will become established and spread is considered to be quite low; most of them are not able to complete the life cycle and establish a stable population outside the station (Chwedorzewska et al. 2013). Although these organisms cannot survive outside at present, they are potential colonisers, which could be established in the future following the climate warming (Convey and Peck 2019). Тhe four cosmopolitan nematode taxa (*E.vulgaris*, *E.filiformis*, *G.villosa* and *P.armatus*) also reported from the Maritime Antarctic are considered to be of non-native origin by Andrássy (1998). Due to the absence or scarcity of data on the morphology of these species, at present, their origin cannot be confirmed. Future studies using an integrated taxonomic approach (i.e. simultaneous molecular and morphological characterisation) of materials obtained from pristine areas may help clarify their status. The gap in knowledge of nematode diversity, both in terms of taxonomy and distribution, is essential when assessing the introduction of non-native species. Nematode species richness in the Maritime Antarctic, which is underestimated (Nielsen et al. 2011b) may be compromised with increasing human impact in Antarctica.

The risk to Antarctic biodiversity is not limited to the transfer of alien species originating from other regions of Earth, but also concerns the transfer of native or endemic species from one part of Antarctica to another where they are not part of the indigenous biota (Convey 2008, Hughes et al. 2019, Hughes et al. 2020). This risk is greater because such species are likely to adapt well to the new location, unlike most non-native species that have been transferred to Antarctica from elsewhere (Convey 2015). The transfer of species across natural biogeographic boundaries can affect endemism in these areas. Antarctica is one of the few regions on the Planet where such boundaries still exist (Convey 2008). The nematode faunas of the Maritime and the Continental Antarctic are characterised by their uniqueness, as no overlap at the species level of the two local faunas exists (Andrássy 1998, Maslen and Convey 2006, Convey et al. 2020). This is indicative of an ancient geographical divide between these areas (Andrássy and Gibson 2007) and led Chown and Convey (2006) to define the Gressitt Line, which is located across the base of the Antarctic Peninsula.

So far, there is no evidence for the transfer and establishment of nematode species from the Continental to the Maritime Antarctic. Some nematological reports have included data on the presence of species that are emblematic of the Continental Antarctic (*Plectusmurrayi* Yeates 1970 and *P.frigophilus* Kirjanova 1958) in the Maritime part, without morphological data (see Velasco-Castrillón et al. (2014a)). In our study, these records are not included as they are most likely due to misidentification.

Regarding the biotope/microhabitat distribution of the species, the incomplete and insufficient data do not allow a definite conclusion, taking into account also the lack of research in the more inaccessible areas of the Antarctic Peninsula and the islands. Most likely the micro biotope distribution pattern is similar to that shown in the study of the nematode fauna of Cape Chelyuskin in the Arctic (Chernov et al. 1979), where species show very low biotopic associations and most of them inhabit all possible microhabitats (i.e. the majority of species are polytopic); this is also a characteristic feature of other groups of organisms in the polar regions (Chernov et al. 1979).

The major life strategy of organisms living in extreme environments is the development of tolerance and plasticity and not lack of competition and specialisation, which is typical of other biomes (Convey 1996, Chernov et al. 2011).

Comparing the two parts of the Antarctic shows that the nematode studies in the Maritime Antarctic are less represented, whereas investigations in the Continental Antarctic have been more intensive. However, the latter are primarily related to ecology (Adams et al. 2014, Velasco-Castrillón et al. 2014a, Velasco-Castrillón et al. 2018) and have identified to date 34 species of soil nematodes (Velasco-Castrillón et al. 2014a). The smaller number of species in the Continental Antarctic is associated with the harsher and more unfavourable environmental conditions. This zone includes ecosystems with the simplest terrestrial fauna on the Planet, where even nematodes are absent (Convey and McInnes 2005, Convey 2017).

The two opposite polar regions of the Earth are unevenly studied with respect to soil nematodes (Peneva et al. 2009, Holovachov 2014a). Despite the fewer taxonomic studies of terrestrial nematodes in the Arctic, 391 species have been recorded there (Holovachov 2014a). Key geographical and ecological features of both regions, such as geological history, climate, landscape, dispersal barriers and vegetation are responsible for the lower nematode diversity in the Antarctic than in the Arctic (Nielsen and Wall 2013).

Studies that include molecular data for the nematodes in the Maritime Antarctic are too rare to provide valuable information regarding nematode diversity, phylogenetics and endemism (Elshishka et al. 2015b, Elshishka et al. 2017, Kagoshima et al. 2019). The taxonomic position of only three Antarctic dorylaimid species, *A.isokaryon*, *P.paradoxus* and *E.signyensis*, was reconsidered on the basis of morphological and molecular characteristics of 18S rDNA (SSU rDNA) and the D2-D3 expansion fragments of 28S rDNA (LSU rDNA) (*Elshishka et al. 2015b, Elshishka et al. 2017*) and two new endemic genera were proposed (*Pararhyssocolpus* and *Enchodeloides*).

To advance the understanding of phylogeny and phylogeography of Antarctic nematodes, studies are required of other genes with higher evolutionary rates than 18S rDNA, such as 28S rDNA, the internal transcribed spacer (ITS in the ribosomal RNA locus) or the mitochondrial cytochrome c oxidase subunit I (COI). These genes should be included in future taxonomic analyses of Antarctic nematodes (Kagoshima et al. 2019).

The application of integrated taxonomy and DNA barcoding will substantially assist in nematode diversity studies, phylogenetics and especially the recognition of cryptic species. Further, comprehensive molecular studies will provide valuable information on the patterns of species distribution and for gaining additional knowledge on evolutionary processes and biogeography of Antarctic nematodes.

The scant studies of polar regions, in particular of the Maritime Antarctic, demand more intensive sampling and research, especially in the territories that have so far remained unexplored, in order to give a clearer and more adequate view of species diversity and trends in their microhabitat and geographical distribution. Therefore, further efforts aiming at targeted and systematic integrative studies are needed.

## Supplementary Material

XML Treatment for Dorylaimida

XML Treatment for Nordiidae

XML Treatment for Enchodeloides signyensis

XML Treatment for Qudsianematidae

XML Treatment for Eudorylaimus coniceps

XML Treatment for Eudorylaimus pseudocarteri

XML Treatment for Eudorylaimus spaulli

XML Treatment for Eudorylaimus verrucosus

XML Treatment for Eudorylaimus cf. carteri

XML Treatment for Pararhyssocolpidae

XML Treatment for Pararhyssocolpus paradoxus

XML Treatment for Dorylaimidae

XML Treatment for Calcaridorylaimus signatus

XML Treatment for Mesodorylaimus antarcticus

XML Treatment for Mesodorylaimus chipevi

XML Treatment for Mesodorylaimus imperator

XML Treatment for Mesodorylaimus masleni

XML Treatment for Aporcelaimidae

XML Treatment for Amblydorylaimus isokaryon

XML Treatment for Aphelenchida

XML Treatment for Aphelenchoididae

XML Treatment for Aphelenchoides haguei

XML Treatment for Aphelenchoides vaughani

XML Treatment for Laimaphelenchus helicosoma

XML Treatment for Alaimida

XML Treatment for Amphidelidae

XML Treatment for Paramphidelus antarcticus

XML Treatment for Monhysterida

XML Treatment for Monhysteridae

XML Treatment for Eumonhystera filiformis

XML Treatment for Eumonhystera vulgaris

XML Treatment for Geomonhystera villosa

XML Treatment for Plectida

XML Treatment for Plectidae

XML Treatment for Plectus antarcticus

XML Treatment for Plectus cf. antarcticus

XML Treatment for Plectus belgicae

XML Treatment for Plectus cf. belgicae

XML Treatment for Plectus insolens

XML Treatment for Plectus tolerans

XML Treatment for Plectus cf. tolerans

XML Treatment for Plectus meridianus

XML Treatment for Plectus cf. meridianus

XML Treatment for Plectus armatus

XML Treatment for Tylenchida

XML Treatment for Psilenchidae

XML Treatment for Antarctenchus hooperi

XML Treatment for Anguinidae

XML Treatment for Ditylenchus parcevivens

XML Treatment for Rhabditida

XML Treatment for Teratocephalidae

XML Treatment for Teratocephalus tilbrooki

XML Treatment for Teratocephalus pseudolirellus

XML Treatment for Teratocephalus rugosus

XML Treatment for Cephalobidae

XML Treatment for Acrobeloides arctowskii

XML Treatment for Cervidellus cf. vexilliger

XML Treatment for Rhabditidae

XML Treatment for Cuticularia firmata

XML Treatment for Rhabditis krylovi

XML Treatment for Rhabditis marina -group

XML Treatment for Peloderidae

XML Treatment for Pelodera teres -group

XML Treatment for Pelodera strongyloides -group

XML Treatment for Pelodera parateres -group

XML Treatment for Mononchida

XML Treatment for Mononchidae

XML Treatment for Coomansus gerlachei

## Figures and Tables

**Table 1.** Distribution of terrestrial nematodes in the Maritime Antarctic. * Taxonomic paper; ** Paper with molecular data; ***Paper with molecular and morphologica data Terrestrial reference sites (SIRS) at Signy Island for long-term monitoring of the various biotic and abiotic components of Antarctic moss-peat communities (for full descriptions, see Tilbrook (1973)). These sites no longer exist and no studies have been done since the late 1980s. SIRS 1 (*Polytrichastrumalpinum* (Hedwig), *Chorisodontiumaciphyllum* (Hook. f. & Wilson) Broth. (60°43.5'S, 45°35.6'W)) SIRS 2 (*Sanioniauncinata* (Hedw.), *Warnstorfiasarmentosa* (Wahlenb.), *Warnstorfialaculosa* (Müll. Hal.), *Cephaloziellavarians* (Gottsche) Steph. (60°43.7'S, 45°36'W)) ^1^ Geographical coordinates according to original paper. ^2^ Geographical coordinates additionally added.

**Nematode species**	**Locality / Coordinates**	**Microhabitat and plant species**	**DNA / Accesion number in GenBank**	**Reference**
*Enchodeloides* *signyensis*	Signy Island (type locality) ^1,2^60°43’S, 45°38’W	*Syntrichiafilaris* (Müll. Hal.) (type habitat); *D.antarctica*; *C.quitensis*		Loof (1975) *
				Maslen (1979b)
		SIRS 1; SIRS 2		Caldwell (1981)
		SIRS 1; SIRS 2		Maslen (1981)
	Alamode Island ^1,2^68°43'S, 67°32'W	*S.uncinata*		Loof (1975)*
				Maslen (1979b)
		Moss		Maslen and Convey (2006)
	Alexander Island ^2^71°0′0″S, 70°0′0″W	Moss, lichen, soil, microbial mat		Maslen and Convey (2006)
	Alexander Island^1^71°52’40’’S, 68°15’57’’W			Convey and Wynn-Williams (2002)
	Blaiklock Island ^1,2^67°33’S, 67°00’W	*P.alpinum*, *Pohlianutans* (Hedw.)		Loof (1975) *
				Maslen (1979b)
	Coronation Island ^1,2^60°38’S, 45°35’W	*D.antarctica*		Loof (1975)*
				Maslen (1979b)
	Charcot Island ^1,2^69°45'S, 75°15'W	Soil, moss clumps, algae, various lichens		Convey et al. (2000)
		Moss, lichen, soil		Maslen and Convey (2006)
	Dream Island ^2^64°44′0″S, 64°14′0″W	Moss mats with green algae		Shishida and Ohyama (1989)*
	Elephant Island ^1,2^61°10’S, 55°14’W	*D.antarctica*; *Polytrichum* sp.		Loof (1975)*
				Maslen (1979b)
	Galindez Island ^1,2^65°15'S, 64°15'W	*D.antarctica*		Loof (1975)*
				Maslen (1979b)
	King George Island ^1^62°09‘32“S, 58°27‘58“W	*D.antarctica*, *C.quitensis*, *Sanionia* sp., *S.filaris*, *Syntrichiamagellanica* (Mont.)		Russell et al. (2014)
	King George Island ^2^62°2′S, 58°21′W	Moist brown soil without vegetation, surrounded by moss	18S rDNA KY881720.1 28S rDNA KY881719.1	Elshishka et al. (2017)***
	Livingston Island ^2^62°36′S, 60°30′W	*D.antarctica*; *D.antarctica*+*S.uncinata*; *D.antarctica*+*S.uncinata*+*C.quitensis*; *P.alpinum*; *S.uncinata*; *Bryum* sp.; *Usnea* sp.+*P.alpinum*; *Cladonia* sp.+*S.uncinata*+*P.alpinum*; *Polytrichumjuniperinum* Hedw.+*S.uncinata*; *S.uncinata*+*Bartramiapatens* Brid.		Peneva et al. (2002)*
				Elshishka et al. (2015a)
		Moss; Soil under moss crust; Soil		Elshishka et al. (2017)*
*Eudorylaimusconiceps*	Signy Island (type locality) ^1,2^60°43’S, 45°38’W	*S.filaris* (type habitat); *Andreaeagainii* Card.; *C.quitensis*; *W.laculosa* and *W.sarmentosa*		Loof (1975)*
		SIRS 1		Maslen (1979b)
		SIRS 2		Maslen (1981)
		*Andreaea* sp.		Pickup (1988)
				Wharton and Block (1993)
	Alexander Island ^2^71°0′0″S, 70°0′0″W	Moss, lichen, soil, microbial mat		Maslen and Convey (2006)
	Ardley Island ^1^62°12‘38“S, 58°56‘40“W			Russell et al. (2014)
	Charcot Island ^1,2^69°45'S, 75°15'W	Soil, moss clumps, algae, various lichens		Convey et al. (2000)
		Moss, lichen, soil		Maslen and Convey (2006)
	Coronation Island ^1,2^60°38’S, 45°35’W	*D.antarctica*		Loof (1975)*
				Maslen (1979b)
	Elephant Island ^1,2^61°10’S, 55°14’W	*D.antarctica*		Loof (1975)*
				Maslen (1979b)
	Emperor Island ^1,2^67°52'S, 68°43'W	*S.uncinata* and *Bryumpseudotriquetrum* (Hedw.)		Loof (1975)*
				Maslen (1979b)
	Guebriant Island ^2^67°48′S, 68°25′W			Maslen (1979b)
	Halfmoon Island ^1^62°35‘45“S, 59°54‘06“W			Russell et al. (2014)
	King George Island ^2^62°2′S, 58°21′W	Mosses		Kito (2009)
	King George Island ^1^62°11‘48“S, 58°59‘28“W; 62°11‘50“S, 58°56‘33“W; 62°11‘53“S, 58°56‘47“W			Russell et al. (2014)
	Leonie Island ^2^67°36′S, 68°21′W	Mixture of soil, moss, lichen, liverworts, algae and cyanobacteria	18S rDNA LC457670.1 LC457669.1 LC457668.1 LC457667.1 LC457666.1 LC457665.1 LC457664.1 LC457663.1 LC457662.1 LC457647.1 LC457646.1 LC457645.1	Kagoshima et al. (2019)**
	Livingston Island ^2^62°36′S, 60°30′W			Elshishka et al. (2015a)
*E.pseudocarteri*	Signy Island (type locality) ^1^60°43’S, 45°38’W	*A.gainii* (type habitat); *W.laculosa* and *W.sarmentosa*; *D.antarctica*		Loof (1975)*
				Maslen (1979b)
		SIRS 1; SIRS 2		Maslen (1981)
		*Andreaea* sp.		Pickup (1988)
				Wharton and Block (1993)
	Adelaide Island ^2^67°15′S, 68°30′W	Moss, lichen		Maslen and Convey (2006)
	Alexander Island ^2^71°0′0″S, 70°0′0″W	Moss, lichen, soil, microbial mat, freshwater		
	Ardley Island ^1^62°12‘38“S, 58°56‘40“W			Russell et al. (2014)
	Charcot Island ^1,2^69°45'S, 75°15'W	Moss, lichen, soil		Maslen and Convey (2006)
	Coronation Island ^1,2^60°38’S, 45°35’W	*D.antarctica*		Loof (1975)*
				Maslen (1979b)
	Elephant Island ^1,2^61°10’S, 55°14’W	*Polytrichum* sp.		Loof (1975)*
				Maslen (1979b)
	King George Island ^2^62°2′S, 58°21′W	Puddle		Tsalolikhin (1989)*
	King George Island ^1^62°09‘32“S, 58°27‘58“W; 62°11‘48“S, 58°59‘28“W; 62°11‘50“S, 58°56‘33“W			Russell et al. (2014)
	Livingston Island ^1^62°39‘14“S, 60°36‘39“W			
*E.spaulli*	Alamode Island (type locality) ^1,2^68°43'S, 67°32'W	Soil around *S.uncinata* (type habitat)		Loof (1975)*
		Moss, lichen, soil, microbial mat		Maslen and Convey (2006)
				Maslen (1979b)
	Adelaide Island ^2^67°15′S, 68°30′W	Moss, lichen		Maslen and Convey (2006)
	Alexander Island ^2^71°0′0″S, 70°0′0″W	Moss, lichen, soil, microbial mat		
	Anchorage Island ^2^67°36′14.01″S, 68°12′32.78″W	Moss, grass, lichen, soil, microbial mat, freshwater		
	Blaiklock Island ^1,2^67°33’S, 67°00’W	*P.alpinum*, *Pohlianutans*		Loof (1975)*
				Maslen (1979b)
	Charcot Island ^1,2^69°45'S, 075°15'W	Soil, moss clumps, algae, various lichens		Convey et al. (2000)
		Moss, lichen, soil		Maslen and Convey (2006)
	Coronation Island ^1,2^60°38'S, 45°35'W	*D.antarctica*		Loof (1975)*
				Maslen (1979b)
	Deception Island ^2^62°58′37″S, 60°39′0″W			Maslen (1979b)
	Elephant Island ^1,2^61°10’S, 55°14’W	*D.antarctica*; *Polytrichum* sp.		Loof (1975)*
				Maslen (1979b)
	Leonie Island ^2^67°36′S, 68°21′W	Moss, grass, lichen, soil, microbial mat, freshwater		Maslen and Convey (2006)
	Limpet Island ^1,2^67°38'S, 68°19'W	*S.uncinata*		Loof (1975)*
				Maslen (1979b)
	Pourquoi pas Island ^2^67°41S, 67°28′W			Maslen (1979b)
	Signy Island ^1,2^60°43’S, 45°38’W	*S.filaris* *B* . *pseudotriquetrum*; *D.antarctica*; *W.laculosa* and *W.sarmentosa*		Loof (1975)*
				Wharton and Block (1993)
		*Andreaea* sp.		Pickup (1988)
				Maslen (1979b)
		SIRS 2		Maslen (1981)
*E.verrucosus*	Elephant Island (type locality) ^1,2^61°10’S, 55°14’W	*D.antarctica* (type habitat)		Loof (1975)*
				Maslen (1979b)
	Adelaide Island ^2^67°15′S, 68°30′W	Moss, lichen, soil, microbial mat		Maslen and Convey (2006)
	Signy Island ^1^60°43’S, 45°38’W			Maslen (1979b)
		SIRS 1; SIRS 2		Maslen (1981)
				Wharton and Block (1993)
Eudorylaimuscf.carteri	Livingston Island ^2^62°36′S, 60°30′W			Elshishka et al. (2015a)
*Pararhyssocolpusparadoxus*	Signy Island (type locality) ^1^60°43’S, 45°38’W	*A.gainii* (type habitat); *S.filaris*		Loof (1975)*
				Maslen (1979b)
		SIRS 1; SIRS 2		Maslen (1981)
	Adelaide Island ^2^67°15′S, 68°30′W	Moss, lichen, soil, microbial mat		Maslen and Convey (2006)
	Adelaide Island ^1^67°34.429'S, 68°07.284'W	*C.varians* and S. *uncinata*		Newsham et al. (2020)
	Anchorage Island ^2^67°36′14.01″S, 68°12′32.78″W	Moss, grass, lichen, soil, microbial mat, freshwater		Maslen and Convey (2006)
	Blaiklock Island ^1,2^67°33’S, 67°00’W			Maslen (1979b)
	Coronation Island ^1,2^60°38'S, 45°35'W	*D.antarctica*		Loof (1975)*
				Maslen (1979b)
	Elephant Island ^1,2^61°10’S, 55°14’W	*S.uncinata*		Loof (1975)*
				Maslen (1979b)
	Galindez Island ^1, 2^65°15'S, 64°15'W	*D.antarctica*		Loof (1975)*
				Maslen (1979b)
	Guebriant Island ^2^67°48′S, 68°25′W			Maslen (1979b)
	Intercurrence Island ^1,2^63°55'S, 61°24'W	*Brachythecium* sp.		Loof (1975)*
				Maslen (1979b)
	King George Island ^2^62°2′S, 58°21′W			Kito (2009)
		Soil	18S rDNA KM092521.1 28S rDNA KM092522.1	Elshishka et al. (2015b)***
	King George Island ^1^62°09‘32“S, 58°27‘58“W; 62°11‘48“S, 58°59‘28“W; 62°11‘50“S, 58°56‘33“W; 62°11‘53“S, 58°56‘47“W			Russell et al. (2014)
	Leonie Island ^2^67°36′S, 68°21′W	Moss, grass, lichen, soil, microbial mat, freshwater		Maslen and Convey (2006)
	Limpet Island ^1,2^67°38'S, 68°19'W	*S.uncinata*		Loof (1975)*
				Maslen (1979b)
	Livingston Island ^2^62°36′S, 60°30′W			Elshishka et al. (2015a)
		*Sanionia* sp.; *C.quitensis*, *D.antarctica*, moss; *D.antarctica*, *C.quitensis*; *D.antarctica*, moss		Elshishka et al. (2015b)*
	Nelson Island ^2^62°18′S, 59°3'W	Moss		Elshishka et al. (2015b)*
*Calcaridorylaimussignatus*	Signy Island (type locality) ^1^60°43’S, 45°38’W	*S.filaris* (type habitat); *B.pseudotriquetrum*; *C.quitensis*; *D.antarctica*; *Prasiolacrispa* (Lightfoot)		Loof (1975)*
				Maslen (1979b)
		SIRS1		Caldwell (1981)
		SIRS 1; SIRS 2		Maslen (1981)
		Soil, moss, lichen, liverworts, algae and cyanobacteria	18S rDNA LC457654.1 LC457653.1 LC457652.1 LC457651.1 LC457650.1 LC457649.1 LC457648.1	Kagoshima et al. (2019)**
	Alamode Island ^2^68°43'S, 67°32'W	Moss, lichen, soil		Maslen and Convey (2006)
	Alexander Island ^2^71°0′0″S, 70°0′0″W	Moss, lichen, soil, microbial mat		
	Anchorage Island ^2^67°36′14.01″S, 68°12′32.78″W	Moss, grass, lichen, soil, microbial mat, freshwater		
	Anvers Island, Biscoe Point ^2^64°49′6.85″S, 63°46′32.29″W	Soil around roots of *D.antarctica*		Shishida and Ohyama (1989)*
	Coronation Island ^1,2^60°38'S, 45°35'W	*D.antarctica*		Loof (1975)*
				Maslen (1979b)
	Dream Island ^2^64°44′0″S, 64°14′0″W	Moss mats with green algae		Shishida and Ohyama (1989)*
	Galindez Island ^1,2^65°15'S, 64°15'W	*D.antarctica*		Loof (1975)*
				Maslen (1979b)
	Leonie Island ^2^67°36′S, 68°21′W	Moss, grass, lichen, soil, microbial mat, freshwater		Maslen and Convey (2006)
*Mesodorylaimusantarcticus*	Livingston Island (type locality) ^1^62°39'22’’S, 60°21'13’’W	*Sanionia* sp. (type habitat); *D.antarctica*; *D.antarctica*-*Polytrichum* sp.; A small moss tuft *Sanionia* sp.; A mix grass-moss spot *D.antarctica*+*Sanionia* sp.		Nedelchev and Peneva (2000)*
	King George Island ^1^62°09‘32“S, 58°27‘58“W; 62°11‘48“S, 58°59‘28“W; 62°11‘50“S, 58°56‘33“W			Russell et al. (2014)
	Neko Harbour, Antarctic Peninsula ^1^64°50‘41“S, 62°31‘53“W			
*M.chipevi*	Livingston Island (type locality) ^1^62°34'48’’S, 60°20'42’’W	*D.antarctica* on the top of flat rock near sea (type habitat); Shallow soil with cover of green algae amongst grass on a rock; Small tuft of *D.antarctica*; *Polytrichum* sp.+*S.uncinata*+*D.antarctica*; A mix grass-moss spot *D.antarctica*+*Sanionia* sp.; A large pure grass spot *D.antarctica*		Nedelchev and Peneva (2000)*
	Livingston Island ^1^62°38'52’’S, 60°22'24’’W	*S.georgico-uncinata* Müll. Hal. + *D.antarctica*		Nedelchev and Peneva (2007)*
	King George Island ^1^62°09‘32“S, 58°27‘58“W			Russell et al. (2014)
*M.imperator*	Emperor Island (type locality) ^1,2^67°52'S, 68°43'W	*S.uncinata* and *B.pseudotriquetrum* (type habitat)		Loof (1975)*
				Maslen (1979b)
	Adelaide Island ^2^67°15′S, 68°30′W	Moss, lichen, soil		Maslen and Convey (2006)
	Alamode Island ^2^68°43'S, 67°32'W	Moss		
	Alexander Island ^2^71°0′0″S, 70°0′0″W	Moss, grass, lichen, soil, microbial mat, freshwater		
	Anchorage Island ^2^67°36′14.01″S, 68°12′32.78″W	Moss, grass, lichen, soil, microbial mat, freshwater		Maslen and Convey (2006)
	Charcot Island ^1,2^69°45'S, 075°15'W	Soil, moss clumps, algae, various lichens		Convey et al. (2000)
	Cone Island ^1,2^67°41'S, 69°10'W	*S.uncinata*		Loof (1975)*
				Maslen (1979b)
	Killinbeck Island ^2^67°34′S, 68°5′W	Moss, lichen, soil		Maslen and Convey (2006)
	Leonie Island ^2^67°36′S, 68°21′W	Moss, grass, lichen, soil, microbial mat, freshwater		
*M.masleni*	Livingston Island (type locality) ^1^62°39'46’’S, 60°23'29’’W	A large area of *D.antarctica* (type habitat); A mix grass-moss spot *D.antarctica* + *Sanionia* sp.		Nedelchev and Peneva (2000)*
*Amblydorylaimusisokaryon*	Elephant Island (type locality) ^1,2^61°10’S, 55°14’W	*D.antarctica* (type habitat); *Polytrichum* sp.		Loof (1975)*
				Maslen (1979b)
	Galindez Island ^1,2^65°15'S, 64°15'W	*D.antarctica*		Loof (1975)*
				Maslen (1979b)
	Intercurrence Island ^1,2^63°55'S, 61°24'W	*Brachythecium* sp.		Loof (1975)*
				Maslen (1979b)
	King George Island ^2^62°2′S, 58°21′W			Kito (2009)
		Soil		Elshishka et al. (2015b)*
	Livingston Island ^2^62°36′S, 60°30′W			Elshishka et al. (2015a)
		Grass spot (*D.antarctica)*; a moss- grass (*D.antarctica*-*Polytrichum* sp.) community; *S.georgico-uncinata* and *D.antarctica*; *C.quitensis* and *D.antarctica*, moss; *D.antarctica* and *C.quitensis*		Elshishka et al. (2015b)*
	Nelson Island ^2^62°18′S, 59°3′W	Moss	18S rDNA KM092519.1 28S rDNA KM092520.1	Elshishka et al. (2015b)***
*Aphelenchoideshaguei*	Signy Island (type locality) ^1^60°43’S, 45°38’W	SIRS 1 (type habitat); SIRS 2		Maslen (1979a)*
		SIRS 1; SIRS 2		Maslen (1981)
	Adelaide Island ^2^67°15′S, 68°30′W	Moss, lichen		Maslen and Convey (2006)
	Adelaide Island ^1^67°34.429'S, 68°07.284'W	*C.varians* and *S.uncinata*		Newsham et al. (2020)
	Alamode Island ^2^68°43'S, 67°32'W	Moss		Maslen and Convey (2006)
	Alexander Island ^2^71°0′0″S, 70°0′0″W	Moss, lichen, soil, microbial mat		
	Anchorage Island ^2^67°36′14.01″S, 68°12′32.78″W	Moss, grass, lichen, soil, microbial mat, freshwater		
	Ardley Island ^1^62°12‘38“S, 58°56‘40“W			Russell et al. (2014)
	Deception Island ^1^62°58‘42“S, 60°33‘29“W			
	King George Island ^1^62°09‘32“S, 58°27‘58“W; 62°11‘48“S, 58°59‘28“W; 62°11‘50“S, 58°56‘33“W; 62°11‘53“S, 58°56‘47“W			Russell et al. (2014)
	Leonie Island ^2^67°36′S, 68°21′W	Moss, grass, lichen, soil, microbial mat, freshwater		Maslen and Convey (2006)
	Livingston Island ^1^62°39‘14“S, 60°36‘39“W	Soil		Russell et al. (2014)
	Livingston Island ^2^62°36′S, 60°30′W			Elshishka et al. (2015a)
*A.vaughani*	Signy Island (type locality) ^1^60°43’S, 45°38’W	SIRS 1; SIRS 2		Maslen (1979a)*
		SIRS 1; SIRS 2		Maslen (1981)
	Alexander Island ^2^71°0′0″S, 70°0′0″W	Moss, lichen, soil, microbial mat, freshwater		Maslen and Convey (2006)
	Livingston Island ^2^62°36′S, 60°30′W			Elshishka et al. (2015a)
*Laimaphelenchushelicosoma*	Signy Island (type locality) ^1^60°43’S, 45°38’W	SIRS 1 (type habitat); SIRS 2		Maslen (1979a)*
		SIRS 1		Maslen (1981)
	Adelaide Island ^2^67°15′S, 68°30′W	Moss, lichen		Maslen and Convey (2006)
	Adelaide Island ^1^67°34.429'S, 68°07.284'W	*C.varians* and *S.uncinata*		Newsham et al. (2020)
	Ardley Island ^1^62°12‘38“S, 58°56‘40“W			Russell et al. (2014)
	King George Island ^1^62°09‘32“S, 58°27‘58“W 62°11‘48“S, 58°59‘28“W 62°11‘50“S, 58°56‘33“W			
				Russell et al. (2014)
	Livingston Island, ^1^62°38'S, 60°20'W	Primitive soil around roots of *D.antarctica*		Peneva and Chipev (1999)*
*Paramphidelusantarcticus*	King George Island (type locality) ^2^62°2′S, 58°21′W	Lichen (type habitat)		Tsalolikhin (1989)*
*Eumonhysterafiliformis*	King George Island ^1^62°09’S, 58°29'W	Thaw ponds, with the bottom inhabited by *W.sarmentosa*		Janiec (1996)
	Livingston Island ^2^62°36′S, 60°30′W			Elshishka et al. (2015a)
*E.vulgaris*	King George Island ^1^62°09’S, 58°29'W	Puddle		Tsalolikhin (1989)*
		Moraine ponds, their shores are inhabited mainly by *S.uncinata*, *W.sarmentosa* and *B.pseudotriquetrum*; Moss banks of *W.sarmentosa* and *W.laculosa*; Thaw ponds, with the bottom inhabited by *W.sarmentosa*; Nearshore ponds, colonised by *W.laculosa* and *W.sarmentosa*		Janiec (1996)
	King George Island ^1^62°09‘32“S, 58°27‘58“W; 62°11‘48“S, 58°59‘28“W; 62°11‘50“S, 58°56‘33“W; 62°11‘53“S, 58°56‘47“W			Russell et al. (2014)
	Livingston Island ^2^62°36′S, 60°30′W			Elshishka et al. (2015a)
*Geomonhysteravillosa*	Coronation Island ^1,2^60°38'S, 45°35'W			Maslen (1979b)
	Ardley Island ^1^62°12‘38“S, 58°56‘40“W			Russell et al. (2014)
	Elephant Islan ^1,2^61°10’S, 55°14’W			Maslen (1979b)
	Galindez Island ^1,2^65°15'S, 64°15'W			
	Intercurrence Island ^1,2^63°55'S, 61°24'W			
	King George Island ^1^62°09’S, 58°29'W	*W.sarmentosa* and *W.laculosa*		Janiec (1996)
	King George Island ^1^62°11‘48“S, 58°59‘28“W 62°11‘50“S, 58°56‘33“W 62°11‘53“S, 58°56‘47“W			Russell et al. (2014)
	Livingston Island ^1^62°39‘14“S, 60°36‘39“W			
	Neko Harbour, Antarctic Peninsula ^1^64°50‘41“S, 62°31‘53“W		
	Signy Island ^1^60°43’S, 45°38’W			Maslen (1979b)
		SIRS 1		Caldwell (1981)
		SIRS 1; SIRS 2		Maslen (1981)
		Mixture of soil, moss, lichen, liverworts, algae and cyanobacteria	18S rDNA LC457677.1 LC457676.1 LC457675.1 LC457674.1 LC457673.1 LC457672.1 LC457671.1	Kagoshima et al. (2019)
*Plectusantarcticus*	Danco Land coast, Beneden Head, Antarctic Peninsula (type locality) ^2^64°46'S, 62°42'W	Freshwater algae (type habitat)		de Man (1904)*
				Maslen (1979b)
		Moss from rock		Andrássy (1998)*
	Adelaide Island ^1^67°34′S, 68°07′W	Moss, lichen, soil, microbial mat		Maslen and Convey (2006)
		*Cephaloziellavarians* (Gottsche)	18S rDNA LC457559.1 LC457558.1 LC457557.1 LC457556.1 LC457555.1 LC457554.1	Kagoshima et al. (2019)**
	Adelaide Island ^1^67°34.429'S, 68°07.284'W	*C.varians* and *S.uncinata*		Newsham et al. (2020)
	Alamode Island ^2^68°43'S, 67°32'W			Spaull (1973a)
				Maslen (1979b)
		Moss		Maslen and Convey (2006)
	Alexander Island, ^1^71°52’40’’S, 68°15’57’’W			Convey and Wynn-Williams (2002)
	Alexander Island ^2^71°0′0″S, 70°0′0″W	Moss, lichen, soil, microbial mat		Maslen and Convey (2006)
	Anchorage Island ^2^67°36′14.01″S, 68°12′32.78″W	Moss, grass, lichen, soil, microbial mat, freshwater		
	Avian Island ^2^67°46"S, 68°54"W			Spaull (1973a)
				Maslen (1979b)
	Blaiklock Island ^1,2^67°33’S, 67°00’W			
	Charcot Island ^1,2^69°45'S, 075°15'W	Soil, moss clumps, algae, various lichens		Convey et al. (2000)
		Moss, lichen, soil		Maslen and Convey (2006)
	Cone Island ^1,2^67°41'S, 69°10'W			Maslen (1979b)
	Coronation Island ^1,2^60°38'S, 45°35'W			
	Deception Island ^2^62°58′37″S, 60°39′0″W	Moss from basalt debris		Andrássy (1998)*
				Maslen (1979b)
	Deception Island ^1^62°58‘43“S, 60°33‘24“W	Only erratic patches of mosses, lichens and algae		Russell et al. (2014)
	Devil Island ^1^63°47‘54“S, 57°17‘24“W	Soil substrates of the very sandy with embedded gravel		
	Elephant Island ^1,2^61°10’S, 55°14’W			Spaull (1973a)
				Maslen (1979b)
		*S.uncinata*		Andrássy (1998)*
	Emperor Island ^2^67°52'S, 68°43'W			Spaull (1973a)
				Maslen (1979b)
	Galindez Island ^1,2^65°15'S, 64°15'W			
	Guebriant Island ^2^67°48′S, 68°25′W			
	Intercurrence Island ^1,2^63°55'S, 61°24'W			Spaull (1973a)
				Maslen (1979b)
	Killingbeck Island ^2^67°34′S, 68°5′W	Moss, lichen, soil		Maslen and Convey (2006)
	King George Island ^2^62°2′S, 58°21′W	Soil around rhizosphere of grasses and under lichen		Tsalolikhin (1989)*
	King George Island ^1^62°09’S, 58°29'W	Moraine ponds, their shores are inhabited mainly by *S.uncinata*, *W.sarmentosa* and *B.pseudotriquetrum*; Moss banks of *W.sarmentosa* and *W.laculosa*		Janiec (1996)
	King George Island ^1^62°09‘32“S, 58°27‘58“W; 62°11‘48“S, 58°59‘28“W	Mosses, lichens, *D.antarctica*		Russell et al. (2014)
	Leonie Island ^2^67°36′S, 68°21′W	Moss, grass, lichen, soil, microbial mat, freshwater		Maslen and Convey (2006)
	Limpet Island ^1,2^67°38'S, 68°19'W			Maslen (1979b)
	Livingston Island ^2^62°36′S, 60°30′W			Elshishka et al. (2015a)
	Pourqoui pas Island ^2^67°41'S, 67°28′W			Maslen (1979b)
	Signy Island ^1^60°43’S, 45°38’W			Spaull (1973a)
				Spaull (1973b)
				Spaull (1973c)
				Maslen (1979b)
		SIRS 1; SIRS 2		Maslen (1981)
		SIRS1; SIRS2		Caldwell (1981)
		*Andreaea* sp.		Pickup (1988)
				Wharton and Block (1993)
		*Acrocladium* sp.; *D.antarctica*		Andrássy (1998)*
Plectuscf.antarcticus	Livingston Island ^2^62°36′S, 60°30′W			Elshishka et al. (2015a)
	Signy Island ^2^60°43’S, 45°36’W	Mixture of soil, moss, lichen, liverworts, algae and cyanobacteria	18S rDNA LC457687.1 LC457686.1	Kagoshima et al. (2019)
*P.belgicae*	Cap Beneden, Danco Land, Antarctic Peninsula (type locality) ^2^64°46''S, 62°42"W	Algae fresh water (type habitat)		de Man (1904)*
	Adelaide Island ^1^67°34′S, 68°07′W	Moss, lichen, soil, microbial mat		Maslen and Convey (2006)
		*C.varians*	18S rDNA LC457565.1 LC457564.1 LC457563.1 LC457562.1 LC457561.1 LC457560.1	Kagoshima et al. (2019)
	Adelaide Island ^1^67°34.429'S, 68°07.284'W	*C.varians* and *S.uncinata*		Newsham et al. (2020)
	Anchorage Island ^2^67°36′14.01″S, 68°12′32.78″W	Moss, grass, lichen, soil, microbial mat, freshwater		Maslen and Convey (2006)
	Charcot Island ^1, 2^69°45'S, 075°15'W	Moss, lichen, soil		
	Elephant Island ^1, 2^61°10’S, 55°14’W	*P.juniperinum*; *S.uncinata*		Andrássy (1998)*
	King George Island ^2^62°2′S, 58°21′W	*B.pseudotriquetrum* and *Bartramiapatens*	18S rDNA LC457638.1 LC457637.1 LC457636.1	Kagoshima et al. (2019)
	Livingston Island ^2^62°36′S, 60°30′W			Elshishka et al. (2015a)
	Rhyolite Island, ^2^69°40′S, 68°35′W	Moss, grass		Maslen and Convey (2006)
	Signy Island ^2^60°43’S, 45°36’W	*Acrocladium* sp.; *Usnea* sp.; SIRS 2		Andrássy (1998)*
Plectuscf.belgicae	Ardley Island ^1^62°12‘38“S, 58°56‘40“W			Russell et al. (2014)
	Deception Island ^1^62°58‘43“S, 60°33‘24“W; 62°58‘42“S, 60°33‘29“W			
	King George Island ^1^62°09‘32“S, 58°27‘58“W; 62°11‘48“S, 58°59‘28“W; 62°11‘50“S, 58°56‘33“W; 62°11‘53“S, 58°56‘47“W			
	Livingston Island ^1^62°39‘14“S, 60°36‘39“W			
	Livingston Island ^2^62°36′S, 60°30′W			Elshishka et al. (2015a)
	Petermann Island ^1^65°10‘29“S, 64°08‘10“W			Russell et al. (2014)
*P.insolens*	Signy Island (type locality) ^2^60°43’S, 45°36’W	Thin soil on rock covered with *Acrocladium* sp. (type habitat); roots of *D.antarctica*		Andrássy (1998)*
	Ardley Island ^1^62°12‘38“S, 58°56‘40“W	Soils, *Sanionia* sp., *W.sarmentosa* and *Andreaearegularis* Müll. Hal.		Russell et al. (2014)
	Livingston Island ^2^62°36′S, 60°30′W			Elshishka et al. (2015a)
*P.tolerans*	Emperor Island (type locality) ^2^67°52’S, 68°43’W	*S.uncinata* (type habitat)		Andrássy (1998)*
	Alexander Island ^2^71°0′0″S, 70°0′0″W	Moss, lichen, soil, microbial mat, freshwater		Maslen and Convey (2006)
	Anchorage Island ^2^67°36′14.01″S, 68°12′32.78″W	Moss, grass, lichen, soil, microbial mat, freshwater		
	Charcot Island ^1,2^69°45'S, 075°15'W	Moss, lichen, soil		
	King George Island ^2^62°2′S, 58°21′W			Kito (2009)
	Leonie Island ^2^67°36′S, 68°21′W	Moss, grass, lichen, soil, microbial mat, freshwater		Maslen and Convey (2006)
	Livingston Island ^2^62°36′S, 60°30′W			Elshishka et al. (2015a)
	Rhyolite Island ^2^69°40′S, 68°35′W	Moss, grass		Maslen and Convey (2006)
Plectuscf.tolerans	Ardley Island ^1^62°12‘38“S, 58°56‘40“W			Russell et al. (2014)
	King George Island ^1^62°09‘32“S, 58°27‘58“W; 62°11‘48“S, 58°59‘28“W; 62°11‘50“S, 58°56‘33“W; 62°11‘53“S, 58°56‘47“W			
	Livingston Island ^1^62°39‘14“S, 60°36‘39“W			
*P.meridianus*	Terra Firma Island (type locality) ^2^68°42′S, 67°32′W	Lichen (type habitat)		Andrássy (1998)*
	Emperor Island ^2^67°52’S, 68°43’W	A carpet of *S.uncinata*		
	Livingston Island ^2^62°36′S, 60°30′W			Elshishka et al. (2015a)
	Signy Island ^2^60°43’S, 45°36’W	Roots of *D.antarctica*		Andrássy (1998)*
Plectuscf.meridianus	Signy Island ^2^60°43’S, 45°36’W	Soil, moss, lichen, liverworts, algae and cyanobacteria	18S rDNA LC457691.1 LC457690.1 LC457689.1 LC457688.1	Kagoshima et al. (2019)
*P.armatus*	Ardley Island ^1^62°12‘38“S, 58°56‘40“W			Russell et al. (2014)
	Coronation Island ^2^60°38'S, 45°35'W			Maslen (1979b)
	Elephant Island ^2^61°10’S, 55°14’W			
	Galindez Island ^2^65°15'S, 64°15'W			
	King George Island ^1^62°11‘48“S, 58°59‘28“W			Russell et al. (2014)
	Livingston Island ^2^62°36′S, 60°30′W			Elshishka et al. (2015a)
	Signy Island ^1^60°43’S, 45°38’W	*D.antarctica*; *C.quitensis*; mosses		Spaull (1973b)
				Spaull (1973c)
				Maslen (1979b)
*Antarctenchushooperi*	Signy Island (type locality) ^1,2^60°43'S, 45°38'W	*A.gainii* (type habitat); *Brachythecium* sp., *Calliergon* sp., *S.filaris*, *Grimmiaantarctici* Card., *Ch.aciphyllum*, *P.juniperinum* and *D.antarctica*		Spaull (1972)*
		*Sanionia* sp.+ *Calliergon* sp.+ *Calliergidium* sp.; *Polytrichum* sp.; *Bryum* sp. + *Syntrichia* sp. + *Andreaea* sp.; *D.antarctica*		Spaull (1973a)
		*A.gainii*, *S.filaris*, *Calliergon*-*Calliergidium*		Spaull (1973b)
				Spaull (1973c)
				Maslen (1979b)
		SIRS 1; SIRS 2		Maslen (1981)
		*S.uncinata*		Caldwell (1981)
				Wharton and Block (1993)
	Coronation Island ^1,2^60°38'S, 45°35'W	*D.antarctica*		Spaull (1972)*
		*D.antarctica*		Spaull (1973a)
				Maslen (1979b)
*Ditylenchusparcevivens*	Signy Island (type locality) ^2^60°43’S, 45°38’W	Fine silt (type habitat)		Andrássy (1998)*
	King George Island ^1^62°09‘32“S, 58°27‘58“W; 62°11‘48“S, 58°59‘28“W; 62°11‘50“S, 58°56‘33“W; 62°11‘53“S, 58°56‘47“W			Russell et al. (2014)
	Livingston Island ^2^62°36′S, 60°30′W			Elshishka et al. (2015a)
*Teratocephalustilbrooki*	Signy Island (type locality) ^1,2^60°43’S, 45°38’W	SIRS 1 (type habitat); SIRS2		Maslen (1979a)*
		‘Swamp’ moss carpets		Maslen (1979b)
		SIRS 1; SIRS 2		Maslen (1981)
		*Andreaea* sp.		Pickup (1988)
				Wharton and Block (1993)
		*Usnea* sp.		Andrássy (1998)*
	Adelaide Island ^2^67°15′S, 68°30′W	Moss, lichen		Maslen and Convey (2006)
	Ardley Island ^1^62°12‘38“S, 58°56‘40“W			Russell et al. (2014)
	Charcot Island ^1, 2^69°45'S, 075°15'W	Moss, lichen, soil		Maslen and Convey (2006)
	Halfmoon Island ^1^62°35‘45“S, 59°54‘06“W	Soil		Russell et al. (2014)
	King George Island ^1^62°09‘32“S, 58°27‘58“W; 62°11‘48“S, 58°59‘28“W; 62°11‘50“S, 58°56‘33“W; 62°11‘53“S, 58°56‘47“W	Soil		
	Leonie Island ^2^67°36′S, 68°21′W	Moss, grass, lichen, soil, microbial mat, freshwater		Maslen and Convey (2006)
*T.pseudolirellus*	Signy Island (type locality) ^1^60°43’S, 45°38’W	*S.filaris* (type habitat)		Maslen (1979a)*
	Alexander Island ^2^71°0′0″S, 70°0′0″W	Moss, lichen, soil, microbial mat, freshwater		Maslen and Convey (2006)
	Charcot Island ^2^69°45'S, 075°15'W	Moss, lichen, soil		
	King George Island ^2^62°2′S, 58°21′W			Kito (2009)
	Livingston Island ^2^62°36′S, 60°30′W			Elshishka et al. (2015a)
*T.rugosus*	Signy Island (type locality) ^2^60°43’S, 45°38’W	SIRS 1; SIRS 2		Maslen (1979a)*
				Maslen (1979b)
		SIRS 1; SIRS 2		Maslen (1981)
	Ardley Island ^1^62°12‘38“S, 58°56‘40“W			Russell et al. (2014)
	Deception Island ^1^62°58‘42“S, 60°33‘29“W			
	Devil Island ^1^63°47‘54“S, 57°17‘24“W			
	Halfmoon Island ^1^62°35‘45“S, 59°54‘06“W			
	King George Island ^1^62°09‘32“S, 58°27‘58“W; 62°11‘48“S, 58°59‘28“W; 62°11‘53“S, 58°56‘47“W			
	Livingston Island ^2^62°36′S, 60°30′W			Elshishka et al. (2015a)
*Acrobeloidesarctowskii*	King George Island (type locality) ^1^58°29'30''W, 61°05'S	Soil around roots of *D.antarctica* (type habitat)		Holovachov and Bostrom (2006)*
	King George Island ^1^62°09‘32“S, 58°27‘58“W			Russell et al. (2014)
	Deception Island ^1^62°58‘43“S, 60°33‘24“W; 62°58‘42“S, 60°33‘29“W	Soil devoid of vegetation or with *P.crispa*		
Cervidelluscf.vexilliger	King George Island ^1^62°09‘32“S, 58°27‘58“W			Russell et al. (2014)
*Cuticulariafirmata*	Signy Island (type locality) ^2^60°43’S, 45°36’W	Fine mud (type habitat); SIRS 2		Andrássy (1998)*
*Rhabditiskrylovi*	King George Island (type locality) ^2^62°2′S, 58°21′W	Flowing lake (type habitat)		Tsalolikhin (1989)*
*Rhabditismarina*-group	Deception Island ^1^62°55‘43“S, 60°40‘48“W			Russell et al. (2014)
*Peloderateres* group	Deception Island ^1^62°58‘43“S, 60°33‘24“W; 62°58‘42“S, 60°33‘29“W			Russell et al. (2014)
	Livingston Island ^1^62°39‘14“S, 60°36‘39“W			
	Livingston Island ^2^62°36′S, 60°30′W			Elshishka et al. (2015a)
	Neko Harbour, Antarctic Peninsula ^1^64°50‘41“S, 62°31‘53“W			Russell et al. (2014)
	Paulet Island ^1^63°34‘30“S, 55°46‘59“W	Ornithogenic soils		
*Peloderastrongyloides* group	Devil Island ^1^63°47‘54“S, 57°17‘24“W			Russell et al. (2014)
	Livingston Island ^2^62°36′S, 60°30′W			Elshishka et al. (2015a)
	Livingston Island ^1^62°39‘14“S, 60°36‘39“W			Russell et al. (2014)
	Neko Harbour, Antarctic Peninsula ^1^64°51‘45“S, 62°26‘47“W; 64°50‘41“S, 62°31‘53“W			
*Peloderaparateres* group	Deception Island ^1^62°58‘42“S, 60°33‘29“W			Russell et al. (2014)
	King George Island ^1^62°09‘32“S, 58°27‘58“W			
*Coomansusgerlachei*	Danco Land coast, Beneden Head Antarctic Peninsula (type locality) ^2^64°46''S, 62°42"W	Algae fresh water (type habitat)		de Man (1904)*
				Maslen (1979b)
				Andrássy (1998)*
	Caleta Cierva, Antarctic Peninsula ^1^64°10’S, 60°57’W	Soil around roots of *D.antarctica*		Chaves (1990)*
	Antarctic Peninsula			Jiménez Guirado et al. (1998)*
	Adelaide Island ^1^67°34.429'S, 68°07.284'W	*C.varians and S. uncinata*		Newsham et al. (2020)
	Anchorage Island ^2^67°36′14.01″S, 68°12′32.78″W	Moss, grass, lichen, soil, microbial mat, freshwater		Maslen and Convey (2006)
	Anvers Island, Biscoe Point ^2^64°49′6.85″S, 63°46′32.29″W	Soil around roots of *D.antarctica*		Shishida and Ohyama (1989)*
	Anvers Island, Old Palmer ^2^64°45′48″S, 64°5′12″W	Moss mats		
	Avian Island ^2^67°46''S, 68°54"W	*Sanionia* sp., *Bryum* sp.		Spaull (1973a)
				Maslen (1979b)
		*S.uncinata*, *B.pseudotriquetrum*		Spaull (1981)
	Coronation Island ^1,2^60°38'S, 45°35'W	*D.antarctica*		Spaull (1973a)
				Maslen (1979b)
	Charcot Island ^1,2^69°45'S, 75°15'W	Soil, moss clumps, algae, various lichens		Convey et al. (2000)
	Deception Island ^2^62°58′37″S, 60°39′0″W	*Sanionia* sp.; *Polytrichum* sp.		Spaull (1973a)
				Maslen (1979b)
		Melting snow moisten mosses in a shingle field		Andrássy (1998)*
	Dream Island ^2^64°44′0″S, 64°14′0″W	Moss mats with green algae		Shishida and Ohyama (1989)*
	Elephant Island ^1,2^61°10’S, 55°14’W	*Brachythecium* sp.; *D.antarctica*; *Sanionia* sp.		Spaull (1973a)
				Maslen (1979b)
		Soil bellow *D.antarctica*		Spaull (1981)
	Galindez Island ^1,2^65°15'S, 64°15'W	*Brachythecium* sp.; *D.antarctica*; *Bryum* sp.; *Sanionia* sp. + *Pohlia* sp.		Spaull (1973a)
				Maslen (1979b)
		*Brachytheciumaustrosalebrosum* (C. Muell.) Par.		Spaull (1981)
	Guebriant Island ^2^67°48′S, 68°25′W	*Brachythecium* sp., *Bryum* sp., *Sanionia* sp.		Spaull (1973a)
				Maslen (1979b)
	Intercurrence Island ^1,2^63°55'S, 61°24'W	*Brachythecium* sp., *Bryum* sp., *Sanionia* sp.		Spaull (1973a)
				Maslen (1979b)
	Halfmoon Island ^1^62°35‘45“S, 59°54‘06“W			Russell et al. (2014)
	King George Island ^1^62°09’S, 58°29'W	Moraine ponds, their shores are inhabited mainly by *S.uncinata*, *W.sarmentosa* and *B.pseudotriquetrum*		Janiec (1996)
	Leonie Island ^2^67°36′S, 68°21′W	Moss, grass, lichen, soil, microbial mat, freshwater		Maslen and Convey (2006)
		Mixture of soil, moss, lichen, liverworts, algae and cyanobacteria	18S rDNA LC457644.1 LC457643.1 LC457642.1 LC457641.1 LC457640.1 LC457639.1	Kagoshima et al. (2019)**
	Limpet Island ^1,2^67°38'S, 68°19'W	*Brachythecium* sp., *Bryum* sp., *Sanionia* sp.		Spaull (1973a)
				Maslen (1979b)
	King George Island ^2^62°2′S, 58°21′W			Kito (2009)
	King George Island ^1^62°09‘32“S, 58°27‘58“W; 62°11‘48“S, 58°59‘28“W; 62°11‘50“S, 58°56‘33“W; 62°11‘53“S, 58°56‘47“W			Russell et al. (2014)
	Litchfield Island ^2^64°46′S, 64°6′W	Decaying moss mats with blue-green algae		Shishida and Ohyama (1989)*
	Livingston Island ^2^62°36′S, 60°30′W	Soil under crisp of green algae; *D.antarctica*-*S.uncinata*; *D.antarctica*, *P.alpinum*		Peneva et al. (1996)*
	Livingston Island ^1^62°39‘14“S, 60°36‘39“W			Russell et al. (2014)
	Livingston Island ^2^62°36′S, 60°30′W			Elshishka et al. (2015a)
	Nelson Island ^2^62°18′S, 59°3W	Moss	18S rDNA KM092523.1 28S rDNA KM092524.1	Elshishka et al. (2015b)**
	Signy Island ^1,2^60°43’S, 45°38’W	*Sanionia* sp.+ *Calliergon* sp.+ *Calliergidium* sp.; *Polytrichum* sp.; *Bryum* sp. + *Syntrichia* sp. +*Andreaea* sp.; *D.antarctica*		Spaull (1973a)
				Spaull (1973b)
				Spaull (1973c)
				Maslen (1979b)
		*S.uncinata* and *W.sarmentosa*		Caldwell (1981)
		SIRS 1; SIRS 2		Maslen (1981)
		*P.crispa* from melt stream		Spaull (1981)
		*Sanionia* sp.		Pickup (1988)
		*P.crispa*; *S.uncinata*		Pickup (1990)
		*Calliergon* sp.		Wharton and Block (1993)
		Mixture of soil, lichen, liverworts, algae and cyanobacteria	18S rDNA LC457661.1 LC457660.1 LC457659.1 LC457658.1 LC457657.1 LC457656.1 LC457655.1	Kagoshima et al. (2019)**
